# Molecular and genetic regulations of fleshy fruit shape and lessons from *Arabidopsis* and rice

**DOI:** 10.1093/hr/uhad108

**Published:** 2023-06-07

**Authors:** Qiang Li, Shuangxia Luo, Liying Zhang, Qian Feng, Lijun Song, Manoj Sapkota, Shuxin Xuan, Yanhua Wang, Jianjun Zhao, Esther van der Knaap, Xueping Chen, Shuxing Shen

**Affiliations:** College of Horticulture, State Key Laboratory of North China Crop Improvement and Regulation, Key Laboratory of Vegetable Germplasm Innovation and Utilization of Hebei, Collaborative Innovation Center of Vegetable Industry in Hebei, Hebei Agricultural University, Baoding, Hebei 071000, China; College of Horticulture, State Key Laboratory of North China Crop Improvement and Regulation, Key Laboratory of Vegetable Germplasm Innovation and Utilization of Hebei, Collaborative Innovation Center of Vegetable Industry in Hebei, Hebei Agricultural University, Baoding, Hebei 071000, China; College of Horticulture, State Key Laboratory of North China Crop Improvement and Regulation, Key Laboratory of Vegetable Germplasm Innovation and Utilization of Hebei, Collaborative Innovation Center of Vegetable Industry in Hebei, Hebei Agricultural University, Baoding, Hebei 071000, China; Center for Applied Genetic Technologies, Institute for Plant Breeding, Genetics and Genomics, Department of Horticulture, University of Georgia, Athens, GA, USA; College of Horticulture, State Key Laboratory of North China Crop Improvement and Regulation, Key Laboratory of Vegetable Germplasm Innovation and Utilization of Hebei, Collaborative Innovation Center of Vegetable Industry in Hebei, Hebei Agricultural University, Baoding, Hebei 071000, China; Center for Applied Genetic Technologies, Institute for Plant Breeding, Genetics and Genomics, Department of Horticulture, University of Georgia, Athens, GA, USA; College of Horticulture, State Key Laboratory of North China Crop Improvement and Regulation, Key Laboratory of Vegetable Germplasm Innovation and Utilization of Hebei, Collaborative Innovation Center of Vegetable Industry in Hebei, Hebei Agricultural University, Baoding, Hebei 071000, China; College of Horticulture, State Key Laboratory of North China Crop Improvement and Regulation, Key Laboratory of Vegetable Germplasm Innovation and Utilization of Hebei, Collaborative Innovation Center of Vegetable Industry in Hebei, Hebei Agricultural University, Baoding, Hebei 071000, China; College of Horticulture, State Key Laboratory of North China Crop Improvement and Regulation, Key Laboratory of Vegetable Germplasm Innovation and Utilization of Hebei, Collaborative Innovation Center of Vegetable Industry in Hebei, Hebei Agricultural University, Baoding, Hebei 071000, China; Center for Applied Genetic Technologies, Institute for Plant Breeding, Genetics and Genomics, Department of Horticulture, University of Georgia, Athens, GA, USA; College of Horticulture, State Key Laboratory of North China Crop Improvement and Regulation, Key Laboratory of Vegetable Germplasm Innovation and Utilization of Hebei, Collaborative Innovation Center of Vegetable Industry in Hebei, Hebei Agricultural University, Baoding, Hebei 071000, China; College of Horticulture, State Key Laboratory of North China Crop Improvement and Regulation, Key Laboratory of Vegetable Germplasm Innovation and Utilization of Hebei, Collaborative Innovation Center of Vegetable Industry in Hebei, Hebei Agricultural University, Baoding, Hebei 071000, China

## Abstract

Fleshy fruit shape is an important external quality trait influencing the usage of fruits and consumer preference. Thus, modification of fruit shape has become one of the major objectives for crop improvement. However, the underlying mechanisms of fruit shape regulation are poorly understood. In this review we summarize recent progress in the genetic basis of fleshy fruit shape regulation using tomato, cucumber, and peach as examples. Comparative analyses suggest that the OFP-TRM (OVATE Family Protein - TONNEAU1 Recruiting Motif) and IQD (IQ67 domain) pathways are probably conserved in regulating fruit shape by primarily modulating cell division patterns across fleshy fruit species. Interestingly, cucumber homologs of *FRUITFULL* (*FUL1*), *CRABS CLAW* (*CRC*) and *1-aminocyclopropane-1-carboxylate synthase 2* (*ACS2*) were found to regulate fruit elongation. We also outline the recent progress in fruit shape regulation mediated by OFP-TRM and IQD pathways in *Arabidopsis* and rice, and propose that the OFP-TRM pathway and IQD pathway coordinate regulate fruit shape through integration of phytohormones, including brassinosteroids, gibberellic acids, and auxin, and microtubule organization. In addition, functional redundancy and divergence of the members of each of the OFP, TRM, and IQD families are also shown. This review provides a general overview of current knowledge in fruit shape regulation and discusses the possible mechanisms that need to be addressed in future studies.

## Introduction

Botanically, fruits are structures of an angiosperm that originate from the gynecium, and play a vital role in seed protection and their dispersion with important evolutionary implications [1]. These structures can be generally classified as fleshy and dry fruits, depending on whether the pericarp is fleshy or dry at the maturity stage [2, 3]. Fleshy fruits are often edible when raw, including the produce of many plants in Solanaceae, Cucurbitaceae, and Rosaceae.

The shape of fleshy fruits largely influences their usage and consumer preference in different geographical locations. For example, large and flat tomatoes are typically used as slicing tomatoes for hamburgers, as they can cover a bun or slice of bread easily. On the other hand, small and cherry tomatoes are mainly consumed raw or used in salads [4, 5]. Moreover, uniformity in fruit shape is highly desirable in mechanical harvesting, as it reduces packaging and transportation cost and increases market value growth. In addition, fruit shape is an important trait selected during domestication and crop improvement [5–7]. The remarkable diversity of fruit shape in cultivated species provides a model system for studying the genetic basis of fruit shape variation [7], thus facilitating efficient manipulation of fruit shape in breeding.

Fruit shape can be affected as early as in the shoot apical meristem (SAM), which develops into the inflorescence meristem (IM) and floral meristems (FMs) after floral induction [8, 9]. Meristem activities and subsequent processes, including gynoecium formation, cell division, and expansion during ovary and fruit development, all contribute to the final fruit shape [10, 11].

The broad diversity of fleshy fruit shapes arises from the growth patterns of adaxial–abaxial, proximal–distal, and medio–lateral axes [7, 10]. Fruit shape is commonly defined by fruit diameter (FD), length (FL), and fruit shape index (FSI), which is the ratio of FL to FD. Tomato, cucumber, and peach have been widely studied and each serves as an excellent model for understanding fruit shape determination in Solanaceae, Cucurbitaceae, and Rosaceae, respectively [5, 7, 12]. Tomato exhibits remarkable diversity in fruit shape, including round, ellipsoid, long, rectangular, flat, heart, long rectangular, obovoid, and oxheart, with FL and FD ranging up to ~10 cm [13–15]. Similarly, cucumber is also well known for its diversity in fruit shape, with fruit length ranging from 5 to 60 cm and diameter from 2 to 5 cm, resulting in a round, cylindrical, long, or extremely long shape [7, 16]. Peach fruit shapes can be simply divided into two groups: round and flat. The FD in the medio-lateral direction is similar between round and flat peaches, whereas fruit height along the proximal–distal axis of round peaches is almost twice as large as that of flat ones [17]. Although many loci related to fruit shape regulation have been identified in tomato, cucumber, and peach, the related mechanisms and comprehensive understanding of fruit shape formation and regulation mediated by the known fruit shape genes remain largely unclear.

Dry fruits can be divided into dehiscent and indehiscent types. The *Arabidopsis* silique and rice grain are examples of these two types, respectively. As a model plant, *Arabidopsis* has contributed significantly to our understanding of silique shape regulation. Meanwhile, rice grain shape has been extensively studied due to its strong association with grain size, crop productivity, and consumer preference. Over the years, significant progress has been made in understanding grain shape regulation in rice, aided by the identification and functional characterization of many genes [18].

In this review, we focus on the OVATE Family Protein - TONNEAU1 Recruiting Motif (OFP-TRM) and IQ67 domain (IQD) pathways regulating fruit shape, with emphasis on tomato, cucumber, and peach. While significant progress has been made in identifying genes regulating fruit shape in *Arabidopsis* and rice, the genetic networks of fleshy-fruit-bearing plants have not been as extensively studied, particularly for the OFP-TRM and IQD pathways. Therefore, summarization of advances in fruit shape regulation mediated by OFP-TRM and IQD pathways in *Arabidopsis* and rice provides valuable insights toward understanding the integration of phytohormones and microtubules in these pathways. The review also discusses the future outlook on research to further understand the mechanisms underlying fruit shape determination.

## Current knowledge of fleshy fruit shape regulation

### Regulators of fruit shape in tomato

Quantitative trait locus (QTL) mapping studies have revealed the genetic basis of tomato fruit shape variation, with at least 13 QTLs identified to date [19–22]. Among them, *fasciated* (*fas*), *locule number* (*lc*), and *excessive number of floral organs* (*eno*) control fruit shape along the medial–lateral axis by mainly increasing locule number. On the other hand, *sun*, *ovate*, *sov1*, *fs8.1*, and *globe* are involved in fruit elongation along the proximal–distal axis by primarily altering the cell division patterning [6, 21, 23].

Locule number significantly affects the size and shape of tomato fruit and is determined during FM development. The ancestor of tomato bears tiny bilocular fruits, while three natural mutations, *fas*, *lc*, and *eno*, gave rise to large and flat tomatoes having eight or more locules [24]. *fas* and *lc* influence locule number in a partially recessive gene action, whereas *eno* acts in a recessive manner [24–27]. *fas* is a partial loss-of-function allele of tomato *CLAVATA3* (*SlCLV3*) resulting from a 294-kb inversion with a breakpoint in the *SlCLV3* promoter. In the wild bilocular *Solanum pimpinellifolium* background, ~50% of the fruits of *fas* near-isogenic lines (NILs) produce three locules [28]. The loss of function of *SlCLV3* produces severely enlarged meristems as well as fasciated flowers and fruits with more locules [28]. The *fas* mutation had a more significant effect on locule number in comparison with the *lc* mutation [25, 29], while the *lc* mutation is much more common in tomato germplasm than the *fas* allele [5]. *lc* is a partial gain-of-function mutation of *WUSCHEL* (*WUS*). The *lc* mutation is caused by two single-nucleotide polymorphisms (SNPs) in a predicted CArG box *cis*-regulatory element downstream of *WUS* [29, 30]. The NILs carrying the wild-type (WT) *lc* allele from Cervil produce fruits with fewer locules (~2.4) in comparison with the NILs that carry the *lc* allele from Levovil (~3.5) [29, 31]. However, in a wild species LA1589 background, *lc* alone had little effect on locule number unless in combination with *fas*. The *eno* mutation is associated with an 85-bp indel upstream of the start codon of *SlENO*, leading to the downregulation of its expression and larger fruits bearing more locules. *SlENO* null mutants develop fasciated flowers and fruits [26]. SlENO was shown to inhibit *SlWUS* expression domains by directly binding to its promoter [26]. Moreover, *eno*, *lc*, and *fas* synergistically regulate locule number [25, 32]. These findings indicated the important role of SlENO in the CLV-WUS module, which regulates FM size and carpel/locule number [30, 33].

**Figure 1:**
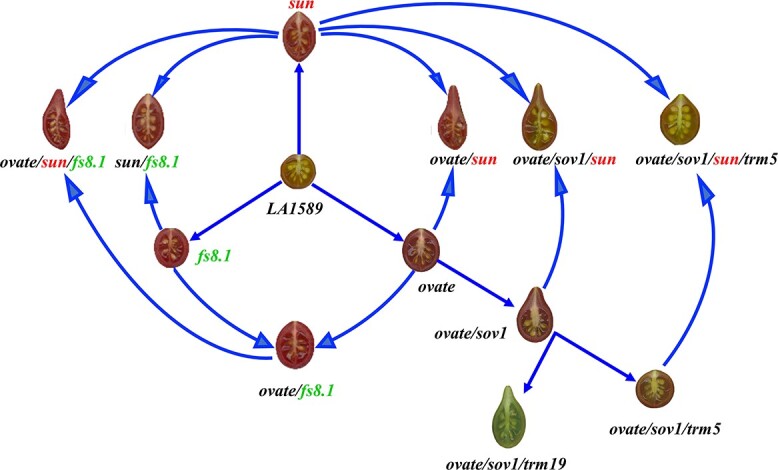
Synergistic effects of fruit elongation mutations in the proximal–distal direction in the LA1589 background. Blue arrows indicate the introduction of mutation. Red, green, and black names of loci represent dominant, partially dominant, and recessive gene action, respectively.

**Figure 2:**
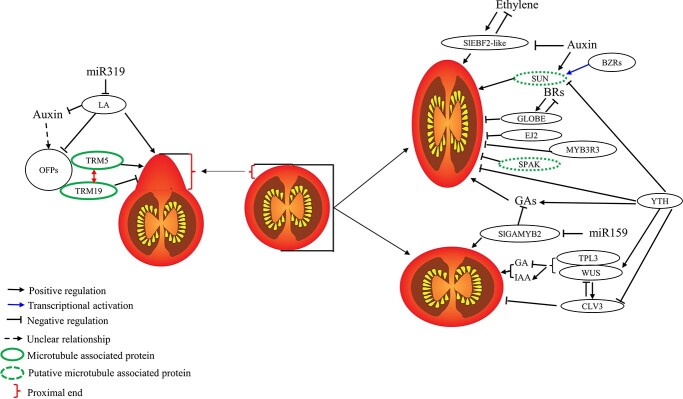
Regulators that control fruit shape in tomato.

On the other hand, the variation of fruit length in tomato is largely explained by *sun*, *ovate*, *sov1*, *fs8.1*, and *globe* (Fig. 1). The *sun* locus is the first identified major locus controlling fruit elongation in tomato. The *sun* locus resulted from a 24.7-kb gene duplication event. This duplication caused *SUN*, an IQD family member, to be driven by the promoter of *DEFENSIN-LIKE1* (*DEFL1*), leading to increased expression of *SUN* on chromosome 7 and elongated fruit [23, 34]. In the LA1589 background, the fruit length of NILs, named LA1589ee, carrying the *sun* locus (~17 mm) was significantly larger than that of WT NILs (~12 mm), and the fruits of LA1589ee were slightly narrower than those of WT. Similarly, transgenic lines overexpressing *SlSUN* produced extremely elongated fruits in the LA1589 background. *SlSUN* controls fruit elongation evenly by increasing ovary wall cell number along the entire longitudinal axis and decreasing ovary wall cell number in the medio-lateral direction (Fig. 2) [23, 34].

The alleles *ovate* and *suppressors of OVATE 1* (*sov1*) are two recessive alleles leading to fruit elongation at the proximal end in tomato [6]. The null mutation *ovate* is a mutation of *OVATE* resulting from a premature stop codon [35]. Although most varieties carrying the *ovate* locus produce pear-shaped or elongated fruits, few such germplasms carry round fruit that results from two *sov* loci [22]. One of them, *sov1*, is caused by a 31-kb deletion upstream of *OVATE Family Protein 20* (*OFP20*), giving rise to reduced expression of *OFP20* [6]. Both OVATE and OFP20 belong to the OFP family with the OVATE domain, which is only found in plants. Overexpressing *OVATE* and *OFP20* in pear-shaped varieties TA503 and Yellow Pear, respectively, changed the fruit shape from pear-shaped to round [6, 35]. In the LA1589 background, while *sov1* alone had no significant impact on fruit elongation, introducing *sov1* to *ovate* NILs leads to pronounced pear-shaped fruits [6], indicating the synergistic interaction between *ovate* and *sov1*. Cellular evaluations of anthesis ovaries in *ovate*, *sov1*, and *ovate*/*sov1* NILs indicated that *ovate* and *sov1* increase cell number along the proximo-distal axis and decrease cell number along the medio-lateral axis at the proximal end, thus conferring pear-shaped fruits [6].

In contrast to *sun*, *ovate*, and *sov1*, which are primarily associated with fruit elongation, *globe* and *fs8.1* affect both fruit weight and shape. *Globe* mainly acts in a recessive manner in controlling globe and flat fruit shapes [21]. The *globe* mutation arose from a thymine (T) insertion in the last exon of *GLOBE*, which is a cytochrome P450 family member and encodes brassinosteroid hydroxylase [21]. The mutation leads to the loss of *GLOBE* function and results in the globe phenotype. Knockout of *GLOBE* using CRISPR/Cas9 causes globe-shaped fruits, confirming the function of the *globe* locus. The study of NILs that differ for the *globe* locus has revealed that *globe* changes fruit weight and shape primarily by regulating fruit elongation at the proximal end of the fruit [21]. The *fs8.1* locus is responsible for up to 27.4% of the FSI variation and acts in a partial dominant manner [36]. *fs8.1* was mapped to a 3.03-Mb region on chromosome 8. *fs8.1* promotes fruit elongation in the proximal–distal direction by increasing cell number. The FL of *fs8.1* NILs was increased by ~12% compared with that of WT NILs [37]. However, the candidate gene of the *fs8.1* locus is still unknown.

Genetic analyses of tomato in the LA1589 background indicated that *sun*, *ovate*, *sov1*, and *fs8.1* exhibit synergistic effects on fruit elongation in the proximal–distal direction (Fig. 1) [38–40]. For example, introducing *sov1* or *sun* into the *ovate* background led to an enhanced effect on pear-shaped fruit [6, 39, 40]. Combination of *sun*/*ovate* with *sov1* or *fs8.1* results in the most elongated tomato fruits, which are more elongated than fruits with each or any two of the three loci [38–40]. Although synergistic interactions were observed among the fruit shape loci, they regulate fruit shape in different patterns. For example, *ovate* and *fs8.1* affect fruit elongation mainly by regulating gynoecium development, whereas *sun* not only regulates gynoecium development but also stimulates fruit elongation after fertilization [38, 40]. Moreover, *ovate* and *sov1* mainly promote cell division at the proximal end, while *fs8.1* and *sun* influence cell division in the entire longitudinal direction [6, 37, 38, 40] (Figs 1 and 2). Further studies are needed to determine whether *globe* genetically interacts with *ovate*, *sov1*, *sun*, and *fs8.1* and the molecular mechanism that ensures coordinated cellular responses.

**Table 1:** Fruit shape genes that have been functionally verified in tomato, cucumber, and peach.

Species	Gene name	Gene ID	Protein category	Functions	Cell proliferation pattern	Reference
Tomato	*LC*/*WUS*	Solyc02g083950	WUS Homeodomain-like superfamily protein	PR of locule number	Cell division and expansion	[25, 28]
	*FAS*/*CLV3*	Solyc11g071380	CLE domain protein	NR of locule number	Cell division and expansion	[25, 28]
	*SUN*	Solyc10g079240	Calmodulin binding protein	PR of fruit elongation	Cell division	[23, 34]
	*OVATE*	Solyc02g085510	OVATE family protein	NR of fruit elongation	Cell division	[6, 35]
	*OFP20*	Solyc10g076180	OVATE family protein	NR of fruit elongation	Cell division	[6]
	*TRM3*/*4*	Solyc03g115000	TONNEAU1 recruiting motif protein	PR of fruit elongation	Cell division	[42]
	*TRM5*	Solyc07g008670	TONNEAU1 recruiting motif protein	PR of fruit elongation	Cell division	[6]
	*TRM17*/*20a*	Solyc06g083660	TONNEAU1 recruiting motif protein	NR of fruit elongation	Cell division	[42]
	*TRM19*	Solyc09g005750	TONNEAU1 recruiting motif protein	NR of fruit elongation	Cell division	[42]
	*GLOBE*	Solyc12g006860	Brassinosteroid hydroxylase	NR of fruit globe phenotype	Cell division and elongation	[21]
	*GAMYB2*	Solyc06g073640	MYB transcription factor	PR of locule number	Cell division and expansion	[52]
	*LA*	Solyc07g062680	TCP transcription factor	PR of fruit elongation	Cell division	[48]
	*SPAK*	Solyc01g097500	NIMA-like kinase	NR of fruit elongation		[63]
	*EJ2*	Solyc03g114840	MADS-box transcription factor	NR of fruit elongation		[64]
	*EBF2-like*	Solyc07g008250	EIN3-binding F-box protein	PR of fruit elongation		[57]
	*MYB3R3*	Solyc09g010820	MYB transcription factor	NR of fruit elongation	Cell division	[66]
	*YTH*	Solyc01g103540	m^6^A reader	PR of locule number		[65]
	*BZR1.5*	Solyc02g071990	BZR1 family transcription factor	PR of fruit elongation	Cell division	[55]
	*BZR1.6*	Solyc03g005990	BZR1 family transcription factor	PR of fruit elongation	Cell division	[55]
	*BZR1.7*	Solyc10g076390	BZR1 family transcription factor	PR of fruit elongation	Cell division	[55]
Cucumber	*FUL1^A^*	Csa1G039910	MADS-box protein	NR of fruit elongation	Cell division and expansion	[76]
	*SUP*	Csa3G141870	Zinc finger protein	PR of fruit elongation	Cell division and expansion	[76]
	*SF1*	Csa2G174140	RING-type E3 ligase	PR of fruit elongation	Cell division	[81]
	*ACS2*	Csa1G580750	1-Aminocyclopropane-1-carboxylate synthase	PR of fruit elongation	Cell division	[81]
	*SF2*	Csa2G337260	Histone deacetylase complex1 (HDC1) protein	PR of fruit elongation	Cell division and expansion	[82]
	*Fnl7.1*	CsGy7G014720	Late embryogenesis abundant (LEA) family protein	PR of fruit neck length	Cell expansion	[91]
	*SUN*	CsaV3_1G039870	Calmodulin binding protein	PR of fruit elongation	Cell division and expansion	[72]
	*HEC1*	Csa4G639900	bHLH family protein	PR of fruit neck length	Cell division	[93]
	*OVATE*	Csa4G038760	OVATE family protein	NR of fruit neck length	Cell division	[93]
	*YUC4*	Csa2G379350	YUCCA (YUC) family protein	PR of fruit neck length	Cell division	[93]
	*CRC^G^*	Csa5G606780	YABBY family protein	PR of fruit elongation	Cell expansion	[79]
	*ARP1*	Csa7G041870	Auxin-responsive protein	PR of fruit elongation	Cell expansion	[79]
	*TRM5*	CsaV3_2G013800	TONNEAU1 recruiting motif protein	NR of fruit elongation	Cell division and expansion	[73]
Peach	*OFP1*	Prupe.6G290900	OVATE family protein	Inducing flat-shaped fruit	Cell elongation	[94, 97]

PR, positive regulator; NR, negative regulator

#### Roles of microtubules in tomato fruit shape regulation

Although the cellular mechanisms underlying fruit shape variation remain largely unknown, recent studies have revealed tight connections between fruit shape variation and microtubules. Microtubule-associated proteins (MAPs) have been found to genetically or physically interact with proteins responsible for fruit shape regulation (Table 1). A prominent example is the OFP-TRM module. The physical interactions between TRMs and OFPs via theTRM M8 motif led to dynamic changes in the localization of the protein complexes, which are proposed to alter the microtubule organization and cell division patterns, ultimately determining fruit shape [6, 41]. In the LA1589 background, while *SlTRM3*/*4* had little effect on fruit shape, knockout of *SlTRM5* resulted in a slightly flatter fruit [42]. The fruit shape of the double mutant of *SlTRM3*/*4* and *SlTRM5* is similar to that of the single null mutant of *SlTRM5* [42]. Introducing the null alleles of *SlTRM3*/*4* or *SlTRM5* into *ovate*/*sov1* NILs partially rescued the pear shape of the fruit, and the combination of both null alleles of *SlTRM3*/*4* and *SlTRM5* in *ovate*/*sov1* NILs resulted in similar FSI to that of WT fruits [6, 42], indicating the additive effects of *SlTRM3*/*4* and *SlTRM5* in regulating fruit elongation (Fig. 2). Interestingly, fruit shape analyses of the null mutants of *SlTRM17*/*20a*, *SlTRM19*, or *SlTRM26a* in the LA1589 background created by CRISPR/Cas9 suggested that *SlTRM17*/*20a* and *SlTRM19* synergistically control fruit elongation, and the small effect of *SlTRM26a* on fruit shape [42]. The null alleles of *SlTRM5* and *SlTRM19* in LA1589 or *ovate*/*sov1* backgrounds were shown to counterbalance each other in regulating fruit elongation, indicating the opposite effects of *SlTRM5* and *SlTRM19* on fruit elongation [42].

The IQD family members in *Arabidopsis* and rice have been found to be associated with microtubules and they might regulate microtubule organization through interactions with calmodulins (CaM), SPIRAL2 (SPR2) and Rho of Plants (ROPs) to impact cell number or shape [43, 44]. Similarly, in tomato, it is possible that SlSUN might regulate fruit elongation by the rearrangement of microtubules through interactions with MAPs [41]. However, further research is required to investigate the potential mechanism.

#### Roles of plant hormones in tomato fruit shape regulation

Plant hormones have been extensively shown to affect cell proliferation and expansion during fruit development and growth [45–47]. Tight links between auxin and fruit shape regulation in tomato have been implicated (Table 1). For example, the application of exogenous auxin to whole plants at anthesis stage using an autospray system resulted in elongated ovaries and fruits with increased cell number at the proximal end along the longitudinal axis, as well as enlarged cell size in most of the tissues in the ovary [38]. The effects of exogenous auxin on fruit elongation were similar to those observed with *sun* and *ovate* in the LA1589 background, indicating that auxin may be involved in the genetic pathways regulating fruit elongation mediated by *sun* and *ovate* [38].

Although exogenous auxin promotes fruit elongation, it increases the expression of OVATE [38]. Furthermore, the auxin level in *ovate* NILs was similar to that in WT NILs [40]. These results indicated that there may not be a linear relationship between *OVATE* expression and auxin levels in controlling fruit shape [38, 40] and auxin may not be directly involved in the pathway mediated by OVATE. This notion was supported by the identification of miRNA-targeted *LANCEOLATE* (*LA*), which was defined as a molecular link between auxin response and OVATE in regulating fruit shape [48] (Fig. 2). *LA* encodes a TEOSINTE BRANCHED1/CYCLOIDEA/PCF (TCP) family protein. The semi-dominant *la* mutant showed elongated fruits resembling *ovate* fruit phenotypes [48]. Moreover, LA directly represses *OVATE* expression and modulates auxin biosynthesis by directly binding to the promoter of *SlYUCCA4* to determine tomato fruit shape [48] (Fig. 2).

Exogenous auxin gave rise to a significantly increased *SUN* expression level, which is consistent with the repression of *Arabidopsis IQD12*, the closest ortholog of tomato *SUN*, upon the inhibition of auxin response [38, 44]. These results indicated that auxin regulates fruit shape likely through direct interaction with SUN [23, 34, 38]. Further biochemical and genetic evidence is needed to confirm this. In addition, several members of the tomato Auxin Response Factor (ARF) family, including SlARF2, SlARF7, and SlARF10, have been shown to impact fruit shape in distinct ways, possibly due to variations in the genetic background of the plants used in the functional analyses [49–51].

Gibberellin (GA), brassinosteroids (BRs) and ethylene were also shown to control fruit shape in tomato (Table 1). A recent study showed that the SlymiR159-SlGAMYB2 pathway regulates fruit shape by modulating GA biosynthesis in tomato [52] (Fig. 2). *SlGAMYB2* is one of the major targets of Sly-miR159 [52]. Transgenic plants with *Sly-miR159* activity suppressed by Short Tandem Target Mimic (STTM159) had larger fruits and decreased FSI with increased fruit locule number, perimeter, and area. Overexpression of *SlGAMYB2* significantly increased fruit weight and locule number compared with WT control fruits and phenocopied the fruit shape change caused by the suppression or loss of function of *Sly-miR159*, while *SlGAMYB2* knockout mutants produce smaller fruits with increased FSI. The reduction of FSI in STTM159 plants as well as plants overexpressing *SlGAMYB2* mainly resulted from the increased FD associated with the increase in locule numbers [52] (Fig. 2). GA_3_ treatment produces elongated fruit, whereas application of the GA inhibitor paclobutrazol results in flatter fruits that are similar to those of the transgenic lines that have downregulation of *SlymiR159* or upregulation of *SlGAMYB2* [52, 53]. Notably, SlGAMYB2 can bind the promoter of the GA biosynthetic gene *SlGA3ox2* and directly repress its transcription, leading to a lower level of active GAs [52]. In addition, the Sly-miR159-SlGAMYB2 module shares similar functions with the CLV-WUS module in regulating tomato locule number. Furthermore, the expressions of *CLV1* and *Fasciated inflorescence* (FIN), two components of the CLV-WUS module, were decreased in STTM159 plants. Further investigation of the interaction between the two modules and the roles of GAs in the two modules will provide new insights into the determination of tomato fruit morphology.

BRs regulate many processes, including fruit development. While many studies have established a role for OFPs in the BR response in rice, the function of BRs in tomato fruit shape regulation is largely unknown. Tomato *GLOBE* encodes a BR hydroxylase that functions in BR catabolism [21, 54], suggesting that GLOBE regulates fruit shape likely through inactivating BRs. Recently, *Brassinazole Resistant 1.5* (*BZR1.5*), *BZR1.6*, and *BZR1.7* were shown to play positive roles in regulating fruit elongation by directly targeting *SUN* to elevate its expression (Fig. 2) [55]. Overexpression of tomato *BZR1.5*, *BZR1.6*, or *BZR1.7* significantly decreased the number of cell layers in the pericarp, leading to decreased pericarp thickness and elongated fruits [55]. While single mutant *bzr1.5*, *bzr1.6*, or *bzr1.7* showed little morphological changes in fruit shape, double mutant *bzr1.5 bzr1.6* and triple mutant *bzr1.5 bzr1.6 bzr1.7* produced flat fruits [55]. The results indicated the functional redundancy of the three *BZR* genes in regulating tomato fruit elongation. Notably, it has been illustrated that the interplay between BR and GA signaling may play a vital role in the fruit shape regulation mediated by OFP20 [56].

Support for the important role of ethylene in the control of fruit shape comes from the evidence that the increased expression of tomato *EIN3-binding F-box protein2-like* (*SlEBF2-like*) leads to elongated fruits with increased FL and decreased FD [57]. *SlEBF2-like* is a close homolog of *Arabidopsis EBF1* and *2*, which have been shown to be negative regulators of the ethylene signaling pathway [57, 58]. However, a change of fruit shape in *Arabidopsis* with overexpression of *EBF1* and *2* was not reported [59], indicating the new functions of *EBF*s in tomato fruit shape regulation.

Although plant hormones play vital roles in fruit shape control, the cellular mechanisms remain elusive. Experimental data from *Arabidopsis* provide evidence that phytohormones, including auxin, GAs, BRs, and ethylene, can regulate cell expansion by modulating microtubule reorientation. While exogenous auxin, GA_4_ and BRs induce transverse microtubule arrays, causing axial growth of hypocotyl cells [60], application of ethylene inhibits root elongation by inducing longitudinal orientation of microtubules [61]. Therefore, it is speculated that auxin, GAs, BRs, and ethylene likely regulate fruit elongation in tomato by modulating microtubule arrays. Identification of the linkers among plant hormones and microtubules will further our understanding of fruit shape regulation. Several lines of evidence in *Arabidopsis* and rice suggest that OFPs and IQDs might be the linkers, which will be discussed below.

In addition to the above-mentioned genes, some other genes affecting tomato fruit shape were also identified (Table 1). For example, SPAK [SP(SELF-PRUNING)-Associated Kinase] belongs to the NEK [NIMA (Never in Mitosis, gene A)-related kinases] family [62]. Downregulation of *SPAK* leads to fruit elongation [63] (Fig. 2). *ej2* is a partial loss-of-function allele of *ENHANCER OF JIONTLESS2* (*EJ2*) resulting from a 564-bp insertion in the fifth intron of the gene. The *ej2* allele arose during domestication and contributes to branched inflorescences and flowers with jointless pedicels. Knockout of *EJ2* resulted in unbranched inflorescences with pear-shaped fruits [64] (Fig. 2). Tomato *YTH* (*YT521B* homology, *SlYTH*) encodes a putative RNA N6-methyladenosine (m^6^A) reader [65]. Interestingly, knockout of *SlYTH* increased expression of *CLV3* and *SUN* and downregulated the expression of *WUS* and the GA biosynthesis pathway, leading to elongated fruit with reduced locule number [65] (Fig. 2). More recently, a tomato *MYB3R3* null mutant was shown to bear elongated fruits, resulting from increased cell numbers along the longitudinal axis at the ovary stage by directly regulating the transcript abundance of the genes involved in the cell-cycle process [66]. However, the mechanisms underlying fruit shape regulation mediated by these genes remains largely unknown.

**Figure 3:**
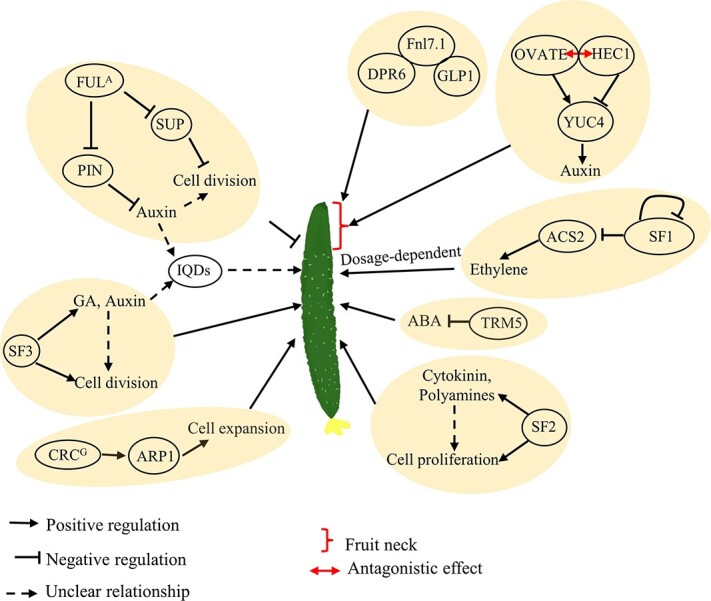
Schematic model of the control of fruit elongation in cucumber

### Regulators of fruit shape in cucumber

Cucumber also varies dramatically in fruit shape [7, 67]. The identification of cucumber fruit shape QTLs and genes [7] has revealed both the common and specific pathways involved in fruit shape regulation, when compared with tomato (Table 1).

#### The OFP-TRM and IQD pathways

At least 30 consensus QTLs related to FL, FD, or FSI have been identified in cucumber [7]. One of the consensus QTLs is *FS3.2*, which has large effects on FL and FD and was detected in at least four populations [68–70]. Two OFP family genes (*CsOFP1b* and *CsOFP13a*) and one IQD family gene (*CsSUN12*) were possible candidates for the *FS3.2* locus [7, 69]. *FS1.2* and *FS2.1* are two major-effect QTLs controlling round fruit shape in cucumber [70, 71]. A 161-bp deletion in the coding region of *CsSUN* was considered to be the underlying mutation of *FS1.2*, causing decreased expression of *CsSUN* in WI7239 harboring round fruits [71]. Genotyping a natural population with the indel marker of the 161-bp deletion indicated that all genotypes bearing elongated or long fruits only had the *CsSUN* allele without the 161-bp deletion. Therefore, *CsSUN* is the most likely candidate to underlie *FS1.2*. Higher expression of *CsSUN* leads to elongated fruit, which is similar to the phenotype found in the *sun* mutant in tomato [34, 71]. Ectopic expression of *CsSUN* in tomato increases the cell number along the longitudinal axis and decreases cell size in the central columellae, leading to elongated fruits [72].

*FS2.1* and *FS1.2* interactively determine the fruit shape by regulating longitudinal and/or radial growth [70, 71]. *FS2.1* was mapped to a 115.0-kb interval on chromosome 2, which contains *CsTRM5*, an ortholog of tomato *TRM5*. Therefore, it is reasonable to consider *CsTRM5* as a best candidate for *FS2.1* [6]. Interestingly, a spherical-fruited mutant, named *qiu*, was identified from the ethyl methane sulfonate (EMS) mutant library in an inbred line bearing short cylindrical fruits. A G/A SNP gave rise to a premature stop codon in *CsTRM5* and was demonstrated to be the causal mutation contributing to the phenotype of the *qiu* mutant [73]. Knocking out *CsTRM5* resulted in decreased FL and increased FD, leading to a change in FSI from 4.9–5.6 to 2.3–2.9. The null alleles of *CsTRM5* decreased fruit shape by enhancing and repressing cell division in the longitudinal and transverse directions of the pericarp, respectively, as well as inhibiting cell expansion in both transverse and longitudinal directions [73]. RNA-seq showed that the genes related to the abscisic acid (ABA) pathway and ABA content were significantly upregulated in *qiu*. Moreover, application of ABA significantly repressed fruit elongation by decreasing cell size in the longitudinal direction [73]. The above results indicate the important role of ABA in the control of cucumber fruit shape mediated by CsTRM5. In addition, ectopic overexpression of *CsOFP11* in *Arabidopsis* leads to shorter and wider siliques, indicating that *CsOFP11* might negatively regulate fruit elongation in cucumber [74].

#### Novel functions of genes for fruit shape regulation in cucumber

Several regulators that have novel roles in cucumber fruit shape regulation have recently been identified (Table 1), indicating that fruit shape regulation in cucumber has its own unique features. For example, tomato MADS-box genes *FRUITFULL* (*FUL1*) and *FUL2* have been shown to regulate fruit ripening [75], whereas *CsFUL1* affects fruit elongation [76]. *CsFUL1^A^* and *CsFUL1^C^* are two natural alleles of *CsFUL1*. *CsFUL1^A^* is a gain-of-function allele of *CsFUL1* and only found in the long-fruited East Asian genotypes. Overexpression of *CsFUL1^A^* resulted in up to ~36.8% decrease in fruit length by inhibiting cell division and expansion. Furthermore, *CsFUL1^A^* directly inhibits the expression of cucumber *SUPERMAN* (*CsSUP*), and the fruits at 10 days after anthesis of CsSUP-RNAi plants were 31–42% shorter than those of control plants. Additionally, *CsFUL1^A^* decreases auxin content in fruits by directly inhibiting the transcript abundances of auxin transporters *PIN-FORMED1* (*PIN1*) and *PIN7* (Fig. 3).

*CRABS CLAW*s (*CRC*s), belonging to the YABBY family genes, have been found to act as major determinants of carpel development in both *Arabidopsis* and tomato [77, 78]. Recently, a non-synonymous SNP (G/A) in *CsCRC* was linked to the fruit shape QTL *FS5.*2 [79]. *CsCRC^A^* was only identified in Xishuangbanna (XIS) cucumbers bearing round or short fruits. The fruits of *CsCRC^A^* NILs were 15–20 cm shorter than those of *CsCRC^G^* NILs. Overexpression of *CsCRC^G^* resulted in a ~9% increase in fruit length, whereas overexpression of *CsCRC^A^* did not show any significant changes in fruit length [79]. *CsCRC^G^* can directly target and upregulate the auxin-responsive gene *CsARP1* [79] (Fig. 3). A study has shown that the knockout of *CsARP1* led to a 31.1% decrease in fruit length due to reduced cell size [79]. Therefore, *CsCRC^G^* promotes cell expansion by directly targeting *CsARP1*, and gives rise to elongated fruits. Interestingly, while the *Arabidopsis crc* null mutant harbors wider and shorter siliques compared with WT, overexpression or downregulation of *SlCRC* in tomato showed no effects on fruit shape [77, 78, 80], indicating the divergence of *CRC* gene functions in plants.

Three EMS-induced short fruit mutations, including *short fruit 1* (*sf1*), *sf2*, and *sf3*, and their corresponding genes have been identified. *sf1* is a loss-of-function mutant resulting from a recessive non-synonymous G-to-A mutation in the eighth exon of *Csa2G174140* [81]. The *sf1* mutant exhibits a short-fruit phenotype with higher expression of *ACS2* and overproduction of ethylene. *SF1* encodes RING-type E3 ligase, which is cucurbit-specific, and targets ACS2 and itself for ubiquitin-dependent degradation to regulate fruit length [81]. Interestingly, although the *acs2* mutant, which resulted from a mutation at residue 33 (G33C), bears short fruits, the ethylene content was significantly reduced. Notably, exogenous application of different concentrations of ethylene to cucumber plants indicated that fruit elongation was stimulated at low concentration (10^−1^ ppm) and repressed at higher concentration (10^1^ ppm). These lines of evidence indicated that SF1 regulates cell division and fruit elongation by controlling ethylene dosage (Fig. 3) [81].

The mutation *sf2* acts in a recessive manner to decrease fruit length by ~50% compared with WT [82]. The short-fruit phenotype is primarily associated with reduced cell proliferation. CRISPR/Cas9-mediated knockout and a complementation test demonstrated that a G-to-A substitution within *SF2*, giving rise to an amino acid change at the 515th residue [Gly (G) to Glu (E)], was the causal mutation contributing to the short-fruit phenotype [82]. *SF2*, encoding a Histone Deacetylase Complex1 (HDC1) protein, promotes histone deacetylation to regulate cell proliferation. The elevated histone deacetylation is related to key genes involved in the biosynthesis and metabolism of polyamines and cytokinin, including *LONELY GUY5* (*LOG5*), *cytokinin oxidase*/*dehydrogenase 7* (*CKX7*) and *S-adenosyl-L-Met decarboxylase* (*SAMDC*) genes (Fig. 3) [82]. The *sf3* mutation changed the fruit length from ~40.0 to ~17.7 cm without influencing fruit diameter [83]. The mutation is likely the result of a non-synonymous C-to-T mutation in the fifth exon of *CsKTN1*, encoding a katanin p60 subunit, and has a semi-dominant effect on fruit length [83]. The decreased indole-3-acetic acid (IAA) and GA levels in *sf3* ovaries and transcriptomic analysis suggested that CsKTN1 controls fruit length likely by modulating the metabolism and signaling of GA and auxin (Fig. 3) [46, 83]. Considering that the orthologs of CsKTN1 in *Arabidopsis* have been shown to be involved in microtubule organization [84], it would be interesting to investigate the roles of CsKTN1 in microtubule organization in cucumber fruit length regulation.

Cucurbits are well known for their plasticity in sex expression [85, 86]. Interestingly, some QTLs controlling fruit shape were shown to co-segregate with the *andromonoecy* locus in cucurbits, leading to fruit shape variation in mapping populations derived from andromonecious parental lines [7, 87]. Ethylene was shown to regulate sex expression and female flower development in cucurbits [88]. *ACS2* encodes an aminocyclopropane-1-carboxylic acid synthase involved in ethylene biosynthesis. Several pieces of evidence suggest that *ACS* genes have a dual function in fruit shape regulation and sex determination. One remarkable example is *CsACS2*, which underlies the *M* locus [89]. In mutant *acs2*, the G/T mutation at the 33rd amino acid residue of *CsACS2* results in reduced ethylene production, leading to hermaphroditic flowers and short fruits, resulting from reduction in cell number along the longitudinal axis [81] (Fig. 3). Recently, an ACS7-dependent regulation of fruit shape was proposed in melon. In this pathway, CmACS7 controls ethylene production to produce elongated fruits by downregulating cell-division-promoting genes, including *E2F-DP*, *OFP*, and *SWI*/*SNF-BAF60*, and upregulating cell-elongation-promoting genes, including *XTH* and *TRM*, to enhance cell elongation [87]. In addition, ectopic expression of *etr1-1*, which is a dominant negative ethylene perception mutant gene, leads to increased ethylene content and elongated fruits under the control of the *CRC* promoter [90]. The mechanisms of how ACS and ethylene affect cell division require further investigation.

Cucumber fruit neck length is highly associated with fruit length. The short fruit neck is a desirable trait and an important breeding objective, especially for long cucumber [91, 92]. CsFnl7.1 was shown to positively control fruit neck length, which primarily modulates cell size by directly interacting with cell expansion proteins, such as dynamin-related protein 6 (CsDRP6) and germin-like protein 1 (CsGLP1) (Fig. 3) [91]. Cucumber *HECATE 1* (*CsHEC1*) showed a high expression level in the fruit neck [93]. Null mutants of *CsHEC1* created by CRISPR/Cas9 had 21–28% decreases in fruit neck length, thus giving rise to short fruits. Interestingly, although overexpression of *CsHEC1* increases fruit neck length by 24–53%, fruit length was comparable between WT and transgenic lines overexpressing *CsHEC1*. Evidence has shown that CsHEC1 can directly target *CsYUC4* to increase its expression, leading to elevated auxin levels and fruit neck length [93]. In addition, CsOVATE negatively regulates fruit neck length by physically interacting with CsHEC1 to weaken the transcriptional activation of *CsYUC4* mediated by CsHEC1 [93]. Unlike CsFnl7.1, which affects fruit neck length by changing cell size, the CsHEC1-CsOVATE module regulates fruit neck length by altering cell number [91, 93]. Given that the OFP-TRM pathway negatively regulates fruit length at the proximal end in tomato, it would be of interest to study the role of TRMs in determining the fruit neck length in cucumber (Fig. 3).

### Regulators of fruit shape in peach

Flat peaches are popular in China due to their low acidity, high sugar content, and association with health and longevity [94]. The flat fruit trait is governed by a single dominant *S* locus mapped using an *F*_2_ population derived from a cross between flat peach and round nectarine [95, 96]. An ~1.7-Mb inversion underlying the *S* locus gives rise to higher expression of *PpOFP1* and flat fruit shape [94, 97, 98]. Overexpression of *PpOFP1* in *Arabidopsis* and tomato resulted in shortened siliques and flat fruits, respectively [94, 97]. Moreover, PpOFP1 can physically interact with PpTRM17 [94], indicating the conservation of the OFP-TRM pathway in tomato and peach. Interestingly, IAA content was significantly increased in round peach compared with flat peach and four genes in the auxin signaling pathway were proposed to be involved in flat fruit shape determination [17], suggesting the important roles of auxin in regulating fruit shape in peach. However, it is yet to be determined whether higher *PpOFP1* expression contributes to lower IAA content in flat peach.

## Lessons from *Arabidopsis* and rice

While our knowledge of the mechanisms underlying fleshy fruit shape is currently fragmented, our understanding of the functions of OFPs, TRMs, and IQDs has been greatly enhanced by their interaction with fundamental regulators of plant development in *Arabidopsis* and rice. These interactions provide valuable insights for elucidating the regulation of fleshy fruit shape.

### The OFP-TRM pathway in *Arabidopsis*

OFPs have been extensively studied in *Arabidopsis* and shown to control organ shape by altering cell division or elongation. The *Arabidopsis* genome contains 19 OFPs, which were classified into three classes based on functional analysis [99, 100]. Plants overexpressing one of the Class I AtOFP genes, including *AtOFP1*, *AtOFP2*, *AtOFP4*, *AtOFP5*, and *AtOFP7*, showed round rosette leaves and short siliques, implying that Class I AtOFPs are associated with inhibition of organ elongation [100–102]. Moreover, AtOFP1 represses cell elongation partially by directly inhibiting *AtGA20ox1* expression [102]. Overexpression of *AtOFP6* or *AtOFP8*, which was designated as a Class II AtOFP gene, resulted in increased thickness of leaves. Plants overexpressing Class III AtOFP genes, including *AtOFP13*, *AtOFP15*, *AtOFP16*, and *AtOFP18*, produced siliques with blunt ends [100, 103]. Overexpression of other AtOFPs did not display any apparent morphological changes [100]. Phylogenetic analysis grouped the 19 AtOFPs into three major clades (C1–C3) [99]. The AtOFP genes belonging to the same functional class also fell into the same clade, indicating their close phylogenetic relationships [99].

The similar phenotypes of the plants overexpressing each AtOFP indicate their overlapping functions in regulating plant development. This notion was further reinforced by the fact that loss of function of single or even two AtOFP genes did not cause any obvious morphological defects [100, 102]. It is of note that the *ofp135* triple mutant and *ofp1235* quadruple mutant produce longer cotyledons and hypocotyls, indicating that they have redundant roles in promoting organ elongation; these mutants display altered microtubule distribution that promotes cell elongation along the longitudinal axis, suggesting that the abnormal microtubule distribution could underlie the mutant organ shapes [101]. AtOFP2 was further shown to be repressed by BR and overexpression of *AtOFP2* inhibits BR-induced cortical microtubule reorientation [101]. However, how BR affects *AtOFP2* expression and microtubule reorientation remains unknown.

In *Arabidopsis*, 34 TRM proteins were identified, and only half of them are putative microtubule-associated proteins [104]. *Arabidopsis* TRM1 and TRM2 and tomato TRM5 belong to the TRM1–5 clade [6]. While SlTRM5 positively regulates fruit elongation by affecting cell division, *Arabidopsis* TRM1 and TRM2 positively regulate the elongation of floral organs by promoting longitudinal cell elongation [41, 105], implying the functional divergence of the TRM1–5 clade in *Arabidopsis* and tomato. Moreover, TRM1 and TON1 directly interact with each other and were found to be localized within non-dividing cells, indicating their important roles in cell elongation [106]. AtOFP1, AtOFP2, AtOFP3, and AtOFP5 proteins were found to interact with TON1, TON2, and TRM1, which are components of the TTP (TON1-TRM-PP2A) complex [101]. Plants overexpressing Class I *AtOFP*s display phenotypes similar to those of *TON2* null mutants, and the quadruple mutant *ofp135 ton2* shows similar phenotypes to the *ton2* single mutant, suggesting that TON2 is essential for the functions of Class I AtOFPs in regulating cell elongation and microtubule reorientation. Thus, AtOFPs probably affect organ shape by modulating microtubule organization and cell elongation, likely through the interaction with the TTP complex [101].

**Figure 4:**
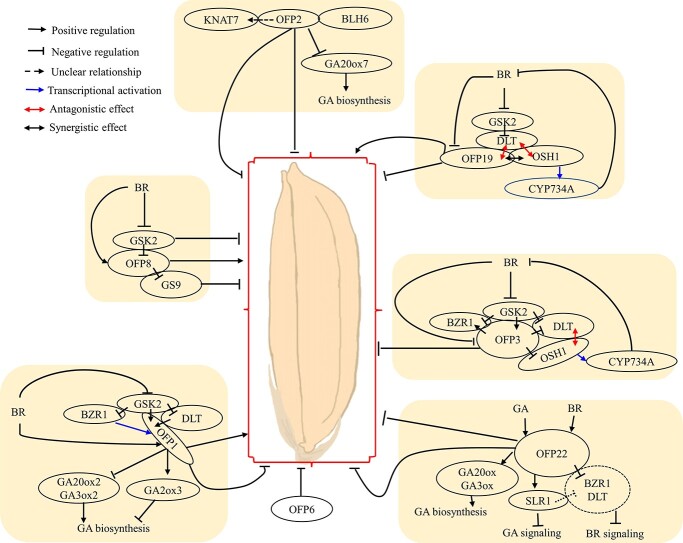
Schematic model of OFPs controlling grain shape in rice.

### The OFP-TRM pathway in rice

The genes responsible for grain length or width regulation are mainly involved in five signaling pathways, including the MAPK signaling pathway, the ubiquitin–proteasome pathway, the G protein signaling pathway, the phytohormone biosynthesis or signaling pathway, and the transcriptional regulation pathway [18, 107]. As the five signaling pathways have been well described in earlier reviews [18, 108, 109], we do not explain them in detail in this review. Instead, our focus is on the OFP-TRM and IQD pathways, which are crucial to grain shape regulation but have received limited attention in existing reviews.

Compared with tomato and *Arabidopsis*, great advances have been made in rice regarding the roles of OFPs in BR signaling in grain shape regulation [18, 108, 109]. Mutants with defects in BR biosynthesis or signaling often display similar phenotypes, including shorter plants and grains, whereas rice plants with increased levels of BRs can produce larger grains, leading to higher yield [18, 110]. Notably, many components in the BR signaling pathway, including rice GSK3/SHAGGY-like kinase 2 (OsGSK2) and Dwarf and Low-Tillering (OsDLT), have been shown to be involved in grain shape regulation [111, 112]. For example, OsGSK2 is a key component that negatively regulates BR signaling. Knockdown of *OsGSK2* resulted in an enhanced BR signaling phenotype, including a ~22% increase in grain length [111, 112]. OsDLT plays a vital role in positively regulating the BR response and signaling, and physically interacts with and is phosphorylated by OsGSK2. Overexpression of *OsDLT* leads to an enhanced BR signaling phenotype with increased grain length and decreased grain thickness and width [112].

The rice genome contains 33 OFP members [99]. Thus far, the biological functions of seven *OFP*s have been reported in rice, and five of them have been shown to regulate grain shape by interacting with primary components of BR signaling (Fig. 4). For example, OsOFP8 was demonstrated to be a positive regulator of BR signaling [111]. Transgenic lines overexpressing *OsOFP8* were hypersensitive to BR treatment and showed increased grain length that is likely caused by enhanced cell division in the longitudinal direction [111, 113]. OsGSK2 physically interacts with and phosphorylates OsOFP8, resulting in the relocalization of OsOFP8 from nucleus to cytoplasm [113]. In addition, OsOFP8 also physically interacts with the Grain Shape gene on chromosome 9 (*GS9*), which affects grain shape by influencing cell division [111]. Notably, the interaction between OsGSK2 and OsOFP8 attenuates the repression effect of OsOFP8 on the transcriptional activity of *GS9* [111]. OsOFP1 is another positive regulator of BR signaling by physically interacting with OsDLT, OsGSK2, and OsBZR1. Overexpression *OsOFP1* plants had enhanced BR responses and increased grain length and decreased grain width. BR positively regulates *OsOFP1* at the transcriptional level through OsBZR1, which directly binds the promoter of *OsOFP1*, as well as by promoting *OsOFP1* stability through the inhibition of OsGSK2 [114]. Moreover, contents of various GA forms were decreased in *OsOFP1*-overexpressing plants with decreased expression of *GA3ox-2* and *GA20ox-2* and elevated expression of *GA2ox-3*, indicating the involvement of OsOFP1 in the BR inhibition of GA synthesis.

OsOFP3, OsOFP19, and OsOFP22 were also revealed to participate in BR signaling, yet all of them play negative roles. Overexpression of *OsOFP3* reduces grain length by repressing cell elongation, which is one of the typical BR-insensitive phenotypes [115]. Further analyses indicated that OsOFP3 physically interacts with many BR-related components, including OsGSK2, OsBZR1, OsDLT, OsOFP1, and *Oryza sativa* homeobox1 (OsOSH1). Unlike OsOFP8, OsBZR1, and OsDLT, which are phosphorylated by OsGSK2 to suppress their activity or alter their subcellular localization [112, 113, 116], OsOFP3 is phosphorylated by OsGSK2 to stabilize the protein [115]. Ectopic expression of *OsOFP19* leads to shorter and wider grains, likely due to the increase in cell number in the periclinal direction [117]. Intriguingly, OsOFP19 interacts with a KNOX protein, OsOSH1, which represses BR biosynthesis by directly upregulating the BR catabolism genes [117, 118]. OsDLT interacts with and functionally antagonizes both OsOFP19 and OsOSH1, forming a functional complex that modulates BR signaling and the cell division pattern in grain shape regulation. Overexpression of *OsOFP22* results in shorter and wider grains by repressing GA and BR signal transduction [119]. These results suggest that the tight links between OFPs and BR signaling are crucial for grain shape formation.

OsOFP2 and OsOFP6 were also shown to control grain shape. Plants overexpressing *OsOFP2* showed decreased grain length and width and increased grain shape index. *GA20ox7* was downregulated in *OsOFP2*-overexpressing plants, which possibly resulted from the interaction of OsOFP2, KNOTTED-LIKE HOMEOBOX OF *ARABIDOPSIS THALIANA* 7 (KNAT7), and BLH6-like proteins, leading to decreased GA content [120]. *OsOFP6* showed abundant expression in the spikelets, and downregulation of *OsOFP6* altered grain shape with significantly reduced grain thickness and width, which is likely caused by repressed cell division by reducing the expression of cell cycle-related genes [121]. Interestingly, *OsOFP6* also regulates the development of lateral roots by affecting polar auxin transport [121]. However, whether *OsOFP6* regulates grain shape by modulating auxin transport requires further evidence.

Several publications on *Arabidopsis* and rice have suggested that the interaction of BR and GA signaling pathways plays a vital role in regulating cell elongation and plant development [122–125]. DELLA proteins, which are the primary negative regulators of the GA signaling pathway, interact with BZR1, a key component that regulates gene expression level in the response to BRs, to inhibit its transcriptional activity [122, 125]. The findings on AtOFP1 and 2, OsOFP1 and 22, and SlOFP20 indicated that the crosstalk between BRs and GAs in regulating fruit shape is likely conserved in plants. The crosstalk of BRs and GAs, especially the interaction between DELLAs and BZR1, in tomato requires further exploration. In addition, it remains to be determined if GA20oxs can be directly regulated by OFPs in tomato and rice, which is similar to the repression of *AtGA20ox1* by AtOFP1 in *Arabidopsis* [102].

In rice, *GRAIN LENGTH ON CHROMOSOME7* (*GL7*), also well known as *GRAIN WIDTH 7* (*GW7*) or *SLENDER GRAIN ON CHROMOSOME 7* (*SLG7*), positively regulates grain length and encodes a protein that is an ortholog of AtTRM1, AtTRM2, and SlTRM5 [126–128]. Interestingly, while GL7/SLG7 promotes cell elongation along the longitudinal axis to regulate grain length, GW7 promotes cell division in the longitudinal direction and inhibits cell division in the transverse direction [104, 127, 128]. However, the conflicting results may be due to the locus interacting within different genetic backgrounds [128]. *OsGW8* represents a major grain shape QTL and encodes the SQUAMOSA Promoter-binding protein-Like 16 (SPL16) transcription factor belonging to the SBP family [129]. OsGW8/SPL16 directly binds to the *GW7* promoter to downregulate *GW7* [127]. Moreover, the expression of *GW8* is negatively regulated by OsmiR156, suggesting an OsmiR156-OsSPL16-GW7 regulatory module that regulates rice grain width and length [127, 129]. These results may be in line with findings in *Arabidopsis*. Downregulation of miR156-targeted *SPL* genes in the *spl8* mutant background results in a shorter gynecium with swollen upper part and narrower basal part [130]. Further studies showed that *SPL8* and the miR156-targeted *SPL*s control fruit shape by influencing auxin signaling and homeostasis [130]. However, whether miR156 and SPLs control fruit shape by regulating TRMs in *Arabidopsis* and tomato remains unknown and requires further investigation.

As with AtTRM1, OsGW7/GL7/SLG7 was also shown to interact with TON1 and PP2A through the M2 and M3 motif, respectively, and target them to cortical microtubules [127], suggesting the interactions among TRMs, TON1 and PP2A are conserved between rice and *Arabidopsis*. However, little is known about the TTP complex in tomato. It would be worthwhile to examine the OFP-TRM interactions in rice, and the interactions among TRM5, TON1, and PP2A in tomato.

### The IQD pathway in *Arabidopsis* and rice

Studies in *Arabidopsis* and rice have shown that IQD proteins, which have also emerged as key regulators of organ shape by mainly affecting cell division, can directly bind microtubules [44, 131]. The *Arabidopsis* IQD family consists of 33 members and most of them showed microtubule localizations [132, 133]. The first IQD member to be studied, IQD1, was identified in *Arabidopsis* and localizes to microtubules and nucleus [134, 135]. AtIQD5, AtIQD6, AtIQD7, and AtIQD8 are four closely related *Arabidopsis* IQD members [136] and all of them labeled cortical microtubules [132]. AtIQD5 regulates pavement cell shape by altering cellulose deposition in the cell wall and microtubule organization [136, 137]. AtIQD6, AtIQD7, and AtIQD8 affect preprophase band formation and division-plane orientation [131, 138]. Interestingly, while all the single mutants of AtIQD5, AtIQD6, AtIQD7, and AtIQD8 displayed indistinguishable phenotypes compared with WT, the *iqd6 iqd7 iqd8* (*iqd678*) triple mutant showed slightly shorter siliques than WT, pointing to the redundant function among the four IQD members. Notably, the *AtIQD5* null mutant showed pavement cells with increased circularity and *iqd8* mutants had increased frequencies of oblique cell walls, indicating the prominent roles of AtIQD5 and AtIQD8 in regulating pavement cell morphogenesis and division-plane control, respectively [131, 136, 137]. Both AtIQD11 and AtIQD16/ABS6 localize to microtubules. Although they belong to different subclades, overexpressing each of them changed the randomly distributed microtubules to oblique arrays, which leads to elongated cells and aerial organs [84, 132, 136]. AtIQD25 localizes at both microtubules and the plasma membrane. While there are no visible morphological changes in *iqd25* mutant compared with WT, plants overexpressing *AtIQD25* showed pavement cells with increased circularity [132].

In rice, a total of 29 IQD members were identified [133]. Rice *GRAIN SIZE ON CHROMOSOME 5* (*GSE5*) underlies the grain width locus *qSW5*/*GW5*. *OsGSE5* encodes an IQD protein that is related to the plasma membrane and plays a negative role in the regulation of grain width by affecting cell proliferation in spikelet hulls. Three indels, a 1212 bp deletion, a 950 bp deletion, and a 367 bp insertion, in the *OsGSE5* promoter in *indica* and *japonica* varieties contribute to grain size diversity and are widely used in rice breeding [139, 140]. OsGSE5 positively regulates the BR signaling pathway by physically interacting with and inhibiting the kinase activity of OsGSK2, leading to enhanced expression of downstream genes that respond to BR [140]. Considering that the IQ67 domain is not required for the interaction and that the fragments interacting with OsGSK2 contain a domain of unknown function, 4005 (DUF4005), it is proposed that only IQDs with DUF4005 are involved in the BR signaling pathway to regulate organ shape in plants. OsIQD14 can directly bind to microtubules through its C-terminal domain. Null mutants of *OsIQD14* produce short and wide grains, while plants overexpressing *OsIQD14* form narrow and long grains. The altered seed shape is mainly due to the alterations of hull cell shape that are likely caused by the modifications of microtubule dynamics [43].

**Figure 5:**
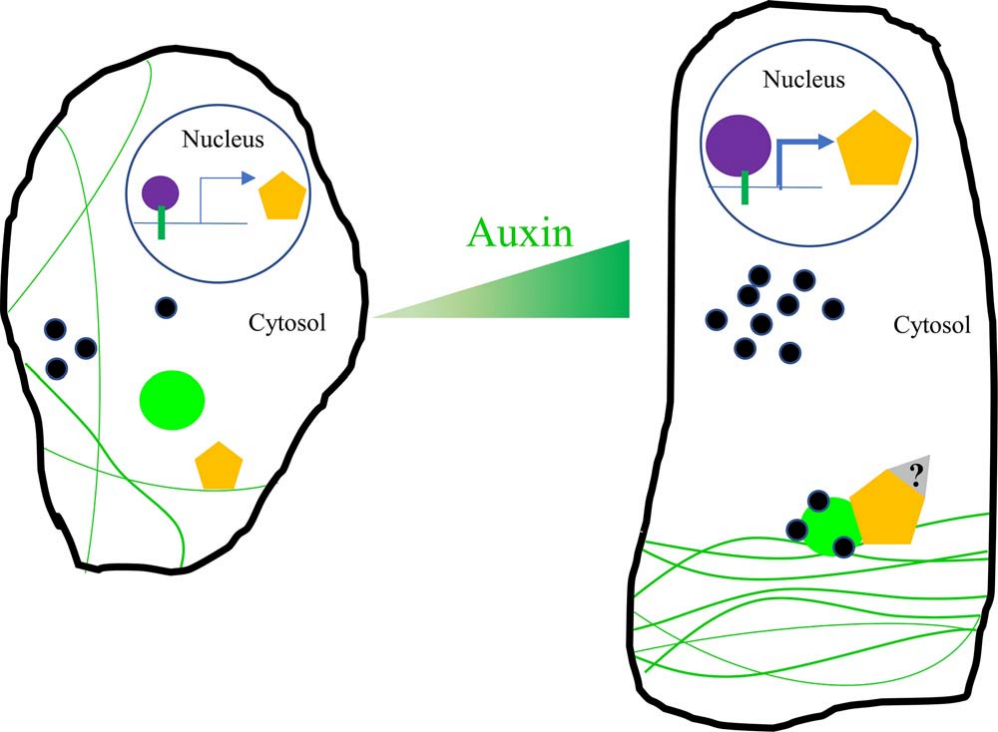
Proposed model of IQDs integrating auxin and calcium signals in the regulation of microtubule orientation and cell shape. On one hand, auxin increases the expression of ARFs, which directly bind to the AuxREs in the promoter of IQDs, thus upregulating the expression of IQDs. On the other hand, auxin also induces an increase in cytosolic Ca^2+^ concentrations. Calcium binds to CaMs and stimulates the physical interaction between CaMs and IQDs, which results in the recruitment of CaMs to microtubules, thus affecting microtubule orientation and cell shape. Black border, plasma membrane; purple circles, ARFs; yellow pentagons, IQDs; green lines, microtubule; green bars, AuxREs; green circles, CaMs; black circles, Ca^2+^; gray triangle, other interacting proteins of IQDs, such as ROP, SPR, and KTN. The size of purple circles and pentagons represents the expression level of ARFs and IQDs, respectively.

Auxin is well known to regulate microtubule dynamics and reorientation, thus affecting cell and organ shape. However, the underlying mechanisms remain elusive. Recent studies in *Arabidopsis* showed that the expression of 13 out of 33 IQDs, including *AtIQD6–8*, *AtIQD11*, and *AtIQD15–18* genes, were altered upon impaired auxin response [44]. The putative ARF binding sites (AuxREs) at the upstream of start codons of *AtIQD15–18* indicate that they might be direct targets of ARF5 in auxin signaling, and the downregulation of AtIQD15 and 18 in the null mutant of ARF5/MONOPTEROS (MP) supports this hypothesis [44, 141] (Fig. 5). Interestingly, auxin can also increase cytosolic Ca^2+^ levels, which could be perceived by Ca^2+^ sensors such as CaM and CaM-like (CML) proteins [142, 143] (Fig. 5). It is well documented in *Arabidopsis* that IQDs directly bind and recruit CaM to microtubules in a calcium-dependent manner [44, 131, 132, 134] (Fig. 5). Thus, IQDs were considered as hubs integrating auxin and calcium signals to regulate microtubule dynamics and reorientation, thereby influencing cell and organ shape [44, 142]. Rice IQD14 is the closest ortholog of the *AtIQD15–18* clade. The rapid induction of *OsIQD14* upon auxin treatment and the interactions of OsIQD14 with CaMs were also observed [43, 144]. In addition, OsGSE5 was also shown to regulate grain shape by directly interacting with calmodulin OsCaM1-1. These results suggested that the integration of calcium signals and auxin by IQDs to manipulate microtubule organization, thus affecting cell and organ shape, is likely conserved between *Arabidopsis* and rice. It is worth noting that the IQDs in tomato and cucumber regulate fruit shape by modulating cell division but not cell expansion, suggesting that IQDs regulate fleshy and dry fruit shape likely through different mechanisms. More research is needed to specify the roles of IQDs in fleshy fruit species in modulating microtubule organization and dynamics.

## Conclusions and perspectives

Fruit shape is a quantitative trait and controlled by numerous loci and complex genetic regulatory networks. However, only a few fruit shape loci have been cloned in fleshy fruit species. Since the cloned loci, such as *sun*, *ovate*, *fs8.1*, and *globe*, have major effects on fruit shape variation, they may overshadow the effects of other fruit shape QTLs, thus hindering the identification of new regulators of fruit shape using classic quantitative assays. To date, fruit shape has been largely characterized by FL, FD, and FSI. Considering that fruit shape is highly dimensional, simplifying the features of fruit shape often leads to a loss of detailed information. For example, even when two fruits have the same fruit length and diameter, it does not mean they will show the same fruit shape. Therefore, modern technologies, including high-resolution mapping populations, next-generation sequencing technologies and genome editing coupled with computational modeling, will be instrumental in accelerating the processes of QTL detection, gene cloning and characterization.

The regulation of fruit elongation mediated by the OFP-TRM pathway and IQD pathway is common in plants. However, many aspects of how the two pathways regulate cell division and expansion remain unclear. Identification of new members involved in the two pathways is important to gain further insights into the molecular mechanisms underlying fruit shape regulation. Considering the synergistic interaction among *ovate*, *ofp20*, *trm5*, and *sun*, it is reasonable to hypothesize that the OFP-TRM and IQD pathways are involved in distinct pathways. Still, both pathways may be involved in the regulation of phytohormone signaling and microtubule reorganization. In particular, recent evidence suggests a plausible link between the OFP-TRM and IQD pathways. First, both TRMs and IQDs are required for preprophase band formation [131, 145]. One of the interacting proteins of IQDs is PHRAGMOPLAST ORIENTING KINESIN 1 (POK1), which is a primary component of the cortical division zone/site (CDZ/CDS) [131, 146]. Interestingly, POK1 was also shown to be regulated by TRMs [145]. Second, TON1 directly interacts with centrin, which is a calcium-binding protein and closely related to CaM, in a calcium-dependent manner [147]. Third, evidence from rice suggests that IQDs and OFPs were found to physically interact with GSK2 to regulate grain shape [111, 114, 115, 140]. Fourth, in *Arabidopsis* the phenotypes of overexpression lines of IQDs are reminiscent of plants with defects in microtubule function, such as the gain-of-function mutant of LONGIFOLIA1/TRM2 and the loss-of-function mutant of TORTIFOLIA 1/SPR2 [105, 132, 148]. Thus, it is possible that OFPs, TRMs, and IQDs coordinately determine organ shape through their involvement in phytohormone pathways and their regulation of microtubule organization and dynamics, which would be an interesting topic for future study. More interestingly, it appears that IQDs and OFPs mediate BR–auxin and BR–GA crosstalk, respectively, through interaction with primary components in the BR signaling pathway to regulate fruit shape [38, 55, 114, 119, 120]. Further epistasis analyses of OFPs, IQDs, and genes involved in BR-, auxin- and GA-related pathways will facilitate a deeper understanding of the interplay of BRs, GAs, and auxin during fruit shape determination. Overall, the identification of novel members of the OFP-TRM and IQD pathways, as well as the understanding of their regulation of phytohormone signaling, microtubule organization and dynamics, and interaction with other pathways, will significantly contribute to the understanding of the molecular mechanisms underlying fruit shape regulation.
